# Current Challenges in *Yersinia* Diagnosis and Treatment

**DOI:** 10.3390/microorganisms13051133

**Published:** 2025-05-15

**Authors:** Bogna Grygiel-Górniak

**Affiliations:** Department of Rheumatology, Rehabilitation and Internal Diseases, Poznan University of Medical Sciences, 61-701 Poznan, Poland; bgrygiel@ump.edu.pl

**Keywords:** *Yersinia enterocolitica*, *Yersinia pseudotuberculosis*, clinical symptoms, diagnosis, treatment

## Abstract

*Yersinia* bacteria (*Yersinia enterocolitica*, *Yersinia pseudotuberculosis*) are commonly found in nature in all climatic zones and are isolated from food (mainly raw pork, unpasteurized milk, or contaminated water), soil, and surface water, rarely from contaminated blood. *Yersinia* infection occurs through sick or asymptomatic carriers and contact with the feces of infected animals. The invasion of specific bacterial serotypes into the host cell is based on the type 3 secretion system (T3SS), which directly introduces many effector proteins (*Yersinia* outer proteins—Yops) into the host cell. The course of yersiniosis can be acute or chronic, with the predominant symptoms of acute enteritis (rarely pseudo-appendicitis or septicemia develops). Clinical and laboratory diagnosis of yersiniosis is difficult. The infection requires confirmation by isolating *Yersinia* bacteria from feces or other biological materials, including lymph nodes, synovial fluid, urine, bile, or blood. The detection of antibodies in blood serum or synovial fluid is useful in the diagnostic process. The treatment of yersiniosis is mainly symptomatic. Uncomplicated infections (diarrhea and abdominal pain) usually do not require antibiotic therapy, which is indicated in severe cases. Surgical intervention is undertaken in the situations of intestinal necrosis. Given the diagnostic and therapeutic difficulties, this review discusses the prevalence of *Y. enterocolitica* and *Y. pseudotuberculosis*, their mechanisms of disease induction (virulence factors and host response), clinical manifestations, diagnostic and preventive methods, and treatment strategies in the context of current knowledge and available recommendations.

## 1. Introduction

*Yersinia*, along with *Shigella* and *Salmonella*, belong to the family *Enterobacteriaceae*. The genus *Yersinia* currently encompasses twenty-six species, three of which are pathogenic for humans: *Y. pestis*, *Y. enterocolitica*, and *Y. pseudotuberculosis* [1]. The infection of *Y. enterocolitica* causes yersiniosis in humans, *Y. pseudotuberculosis* is responsible for Far East scarlet-like feverdevelopment, and *Y. pestis* leads to plague [2]. Human yersinioses most often occur as sporadic cases, but clusters of cases or outbreaks have also been described [3,4,5,6,7].

Since the most common human enteropathogens are *Y. enterocolitica* and *Y. pseudotuberculosis*, which both have a worldwide distribution [2], only these will be discussed in this article.

Bacilli from the genus *Yersinia* have enterobacterial common antigen (ECA) in their cell envelope, which allows them to be classified as Enterobacteriaceae. Both pathogens (*Y. enterocolitica* and *Y. pseudotuberculosis*) are foodborne diseases transmitted by the fecal—oral route. Unfortunately, they have become more common in recent years due to transmission by humans and farm animals; however, *Y. enterocolitica* is detected more often than *Y. pseudotuberculosis*. *Yersinia* can grow over a wide range of temperatures (0–42 °C), which increases their virulence potential. Specifically, the nutritional infestation and infection development of *Y. enterocolitica* after serotype O3-contaminated blood was stored at 4 °C for transfusion has been described in the literature [8]. Typically, enteric bacteria cause self-limited acute infections that spread from the intestine to the mesenteric lymph nodes. They cause infections mainly in children; however, severe illnesses and chronic conditions can also occur, especially in immunocompromised individuals [9,10,11,12,13,14].

Recently, many studies have broadly discussed bacterial virulence, their rapid reproduction, and the possibility of various diagnostic methods allowing for faster bacteria detection and effective therapeutic management [15,16,17,18]. Unfortunately, such a management model is not always possible in clinical practice. The diagnosis is usually delayed due to late infection reporting by patients, a lack of access to appropriate diagnostic methods, and often excessive or too short treatment. Therefore, this review discusses the prevalence of *Yersinia*, pathomechanisms, different clinical variants of the disease, diagnostic and prophylactic methods, and current recommendations for treatment. The current status of the surveillance, detection, and prevention of yersiniosis is also described.

## 2. Materials and Methods

This review article was formed from a PubMed and Google Scholar literature search conducted until October 2024 that included meta-analyses, case reports, case studies, systematic reviews, randomized controlled trials (RCTs), and prospective and retrospective cohorts. Out of these, a stark emphasis was placed on systematic reviews, meta-analyses, and cohorts, while individual case studies, case reports, and RCTs were mainly referred to.

The search words used to include the manuscripts were as follows: *Y. enterocolitica*, *Y. pseudotuberculosis*, epidemiology, clinical symptoms, diagnosis, pathomechanism, reservoir, transmission, and treatment. Conjunction words such as AND and OR were also used to maximize the search further. From the manuscripts, only English manuscripts were included. The literature search included articles from 2014 onwards. Other relevant articles aligned with the authors’ aims for the review were included outside the established timeframe to include key information.

## 3. Historical Background

*Yersinia* causes intestinal and extraintestinal symptoms. The genus name *Yersinia* comes from the etiological agent of plague, related to its discovery in 1894 during the “Black Death” epidemic in Hong Kong, and was independently discovered by two researchers, French bacteriologist Alexander Yersin and Japanese physician Shibasaburo Kitasato. The isolated Gram-negative rod was named *Yersinia* pestis [19]. However, *Y. pestis* evolved from *Y. pseudotuberculosis* 1500–20,000 years ago, before the first known plague pandemics in humans [20]. Later, with the development of many microbiological methods, scientists noticed that similar Gram-negative rods (such as *Y. pseudotuberculosis* and *Y. enterocolitica*) were isolated before Y. pestis was classified into the genus *Yersinia* [21]. However, the history of the discovery of individual Yersinia species and their taxonomy was long and convoluted. In 1939, at the New York State Department of Health, Schleifstein and Coleman identified a new species of bacteria from five bacterial isolates that they believed resembled *Actinobacillus lignieri* and *Pasteurella pseudotuberculosis*. The bacteria were highly virulent to mice, and because three strains originated from intestinal contents, they proposed the name *Bacterium enterocoliticum* [22]. In 1994, van Longhem proposed the genus name *Yersinia* (from the family Enterobacteriaceae), in honor of the French bacteriologist Alexander Yersin [23]. It was not until 20 years later that the Danish microbiologist Wilhelm Frederiksen established the taxonomy of *Yersinia enterocolitica*, classifying the discovered bacteria in the genus Yersinia and changing its name to *Y. enterocolitica* [24]. The DNA–DNA hybridization technique used by Brenner et al. in 1976 showed that both indole-positive and indole-negative strains of *Y. enterocolitica* are closely related (with a DNA hybridization relatedness of 79 to 100%) and can be considered as one species [25]. In 1981, three groups of bacteria were separated from *Y. enterocolitica* (*Y. intermedia*, *Y. frederiksenii*, *Y. kristensenii*) [26,27]. The biochemical heterogeneity observed by Nilehn in 1969 justified the creation of five biogroups (biotypes) [28]. The biogrouping described then was modified by Wauters et al. in 1987, who distinguished six biogroups of *Y. enterocolitica* [29]. Two of these biogroups (biogroups 3A and 3B) were subsequently determined to be speciated and designated as the species *Y. bercovieri* and *Y. mollaretii* (Wauters, 1988) [30].

## 4. Epidemiology of *Yersinia* Infection

The infections of *Yersinia* spp. have been reported from all continents but are most common in Europe [31]. In developed countries, *Y. enterocolitica* is a frequent cause of gastroenteritis. Interestingly, it is also the third most common bacterial cause of gastrointestinal infections in European populations [32].

The prevalence of yersiniosis is higher in northeastern Europe than in other European countries [33]. For example, the incidence rate of *Y. enterocolitica* in Sweden is 2.27–6.18 cases per 100,000 inhabitants [34]. Yersiniosis is also reported in Poland [35] and a decreasing trend in the incidence of yersiniosis has been observed since 2009. In 2018–2020, a total of 542 cases of yersiniosis were recorded (456 intestinal and 86 extraintestinal). In 2020, a sharp decrease in the number of yersiniosis infections was observed compared to previous years, resulting from the introduction of many restrictions to reduce the spread of the SARS-CoV-2 virus, which also affected the number of yersiniosis infections [36]. In the USA, *Y. enterocolitica* causes about 116,716 human infections annually [37]. The Centers for Disease Control and Prevention in the USA assessed that *Y. enterocolitica* is responsible for 640 hospitalizations and 35 deaths yearly [38]. The incidence of yersiniosis is also monitored in developing countries; however, the exact prevalence of infections is unknown in Africa and the Middle East due to insufficient diagnostics [39].

The decreasing EU trend for confirmed yersiniosis cases since 2008 stabilized during 2013–2017. In 2017, yersiniosis was the third most reported zoonosis in the European Union (n = 6823 confirmed cases reported by twenty-six MS (member states); the notification rate was 1.77 cases per 100,000 population, which was 2.8% lower than in 2016). The highest country-specific notification rates were in northeastern Europe. The decreasing trend of human yersiniosis was observed in the EU/EEA from 2008 to 2017, but the trend did not show any significant increase or decrease in the past five years (2013–2017). The most common bioserotype was 4/O:3, followed by 2/O:9 and three fatal cases were reported among the 4467 confirmed yersiniosis cases for which this information was available. Of the 12 identified outbreaks, 11 were caused by *Y. enterocolitica* (the large outbreak consisted of 80 patients in Denmark) [40].

Currently, in the European Union, yersiniosis is the third most common foodborne zoonosis in humans (7919 confirmed cases of yersiniosis in humans in 2022 and 8738 in 2023). According to the European Food Safety Authority (EFSA) and the European Centre for Disease Prevention and Control (ECDC, 2024), a stable trend in incidence was observed between 2018 and 2023. Most cases of yersiniosis are caused by *Y. enterocolitica*, although sporadic outbreaks caused by *Y. pseudotuberculosis* occur. For example, in 2022, *Yersinia enterocolitica* was the species reported in the majority (98.7%) of human cases, while *Y. pseudotuberculosis* was reported in only 1.3% of human cases [41]. The *Y. pseudotuberculosis* infection is rare, and usually, sporadic cases are reported. Nevertheless, outbreaks have been reported in Japan [42], Canada [43], Europe [6], Russia [3], and New Zealand [4]. In Europe, the disease is usually a self-limiting acute enteritis that spreads to the mesenteric lymph nodes [14,44,45]. However, outbreaks of *Y. pseudotuberculosis* infections can be severe and are characterized by systemic inflammatory symptoms called Far Eastern scarlet fever (FESLF), mainly reported in Russia and Japan [46]. FESLF is a rare, severe inflammatory disease that, unfortunately, is often lethal [46]. FESFL, also known as Izumi fever, has clinical features resembling those of Kawasaki disease and mainly develops among young children, adolescents, and young adults in Japan. The incidence of this disease has markedly decreased because of improved sanitation [47].

## 5. Pathomechanisms of Yersiniosis Infection

Enteric yersiniosis is a disease transmitted through the fecal–oral route [48]. Pathogenic strains are detected in the raw intestines of infected animals, unpasteurized milk, or contaminated water. Groups exposed to yersiniosis include butchers and farmers. Infections rarely occur through direct contact with animals or contaminated blood during a transfusion [49]. The predominant strain of *Y. enterocolitica* causing infections in humans is serogroup O:3; however, other serogroups (O:8 and O:9) are also responsible for clinical symptoms in European countries and the United States [50].

*Y. pseudotuberculosis* is classified into serotypes O1 to O14 [51]. *Y. pseudotuberculosis* O:1 was isolated from 39 stool specimens and five (42%) of twelve soil samples [52]. The European strains of *Y. pseudotuberculosis* serotype O1 usually exhibit a pathogenicity island called the high-pathogenicity island (HPI) on the chromosome of *Yersinia* spp. [53]. The Far Eastern strains of *Y. pseudotuberculosis* produce a superantigenic toxin called *Y. pseudotuberculosis*-derived mitogen (YPM) encoded by a gene on the chromosome [54]. Most serotypes O1 to O5 are pathogenic and have been detected in Europe and the Far East. Serotypes O6 to O14 have been isolated only from wild animals in the Far East (Russia, Korea, mainland China, and Japan) but never from clinical samples [55].

## 6. Animal Reservoirs of *Yersinia*

*Yersinia* spp. are foodborne pathogens [29,37]. The most common *Yersinia* species (*Y. enterocolitica*, *Y. pseudotuberculosis*) are transmitted via the fecal–oral route. Human infections most often occur after the consumption of contaminated food or water, and less frequently by direct contact with infected animals [14]. The main risk of *Yersinia* infection is associated with food sources of animal origin, particularly pigs and pork products [56,57,58,59]. In contrast, the main environments and reservoirs of *Yersinia* include soil, water, and various animals [14]. Pigs are asymptomatic carriers of the bacteria in their tonsils and gastrointestinal tract and excrete the enteropathogen into the environment via feces. Pork contamination often occurs during the evisceration of pigs during slaughter [60].

### 6.1. Reservoirs of Yersinia enterocolitica

*Y. enterocolitica* is a ubiquitous microorganism, but most isolates from asymptomatic carriers, food, and the environment are not pathogenic [48]. The meta-analysis from Guillier highlighted food exposures as the significant risk factors of yersiniosis. It confirmed that the predominant reservoirs are pigs (the consumption of raw or undercooked pork, occupational contact with pigs). Infection can also be caused by untreated drinking water. Thus, food and water are significantly associated with sporadic *Y. enterocolitica* infections [61].

Interestingly, *Y. enterocolitica* 4/O:3 is the most common cause of sporadic human yersiniosis caused by contaminated pork meat in Finland and Germany [56]. Italian data have shown that the pathogenic biotypes of *Y. enterocolitica* are uncommon in foods and are mainly isolated from animal sources (including pork and poultry meat and their derivatives) [57]. Chinese data indicated that domestic farm dogs can also transmit *Yersinia* infection [62]. Similarly, the suspected route of transmission is through contact with domestic animals (Sweden, Denmark, China) [59,63]. *Y. enterocolitica* has been detected in unpasteurized milk and cow feces in the United Kingdom and the United States. However, the prevalence in cows is lower than in pigs [62,64]. Pathogenetic serotypes have also been detected in sheep and goats [62,64]. A Swiss study showed that *Y. enterocolitica* and *Y. pseudotuberculosis* are also commonly found in wild boars, with a higher incidence in younger individuals [65].

Yersiniosis can also be transmitted by wild animals such as brown hares, bats, and wild rodents [66,67,68] *Y. enterocolitica* has been isolated from the stool of wild boars, red deer, and roe deer (the *Ail* gene was isolated, suggesting a virulence gene for *Y. enterocolitica*) [69]. In Poland, *Y. enterocolitica* is frequently isolated from game animals, which poses a risk of spreading to other animal species and humans [70]. In Spain, wild boars are very common reservoirs of *Y. enterocolitica* and *Y. pseudotuberculosis* [71].

### 6.2. Reservoirs of Yersinia pseudotuberculosis

The main reservoirs of *Y. pseudotuberculosis* are wild mammals and birds, but the bacteria have also been detected in contaminated food such as iceberg lettuce [72], carrots [73], or raw milk [74].

*Y. pseudotuberculosis* infection (a foodborne pathogen) is not as common as *Y. enterocolitica*, and there are only limited data on its occurrence. This fact may be the reason for the poor awareness of *Y. pseudotuberculosis* as a causative agent of gastroenteritis. In addition, reservoirs of *Y. pseudotuberculosis* other than *Y. enterocolitica* have been reported. Many difficulties are also associated with the bacterial culture detection of this species, which may cause diagnostic difficulties. The reservoirs of *Y. pseudotuberculosis* are mainly wild mammals (especially rodents, lagomorphs, and wild boars) and birds (Table 1). The pathogen can enter the food chain, and the consumption of contaminated food causes disease outbreaks. Reported dietary sources include iceberg lettuce [72], carrots [73], and raw milk [74]. *Y. pseudotuberculosis* replicates in the intestinal lumen, invades the small intestine through Peyer’s patches to initiate dissemination, and spreads to mesenteric lymph nodes [75]. This pathogen has also been detected in the spleen and liver [76].

*Y. pseudotuberculosis* has a more diverse host spectrum, including human pathogens and infected farm, domestic, and wild animals [62,65,71,77,78,79]. Approximately 0.6 to 5 cases of *Y. pseudotuberculosis* per 100,000 inhabitants have been reported in humans (data from Finland) [77]. *Y. pseudotuberculosis* has been isolated from asymptomatic porcine tonsils and intestinal contents. Therefore, infected pigs could not be immediately identified during slaughter. The prevalence of *Y. pseudotuberculosis* in fattening pigs’ tonsils and intestinal contents ranged from 0.03% to 6% [77,80]. This pathogen has been isolated not only from pork but also from wild game meat [81,82]. *Y. pseudotuberculosis* occurs in 20% of wild boars in Sweden and Switzerland (isolates from tonsil tissue) [65,78]. Thus, both wild game meat (wild boars) and outdoor pig farming can be reservoirs for *this pathogen* (Spanish, Swedish, and German data) [71,78,79]. In Finland, outbreaks of *Y. pseudotuberculosis* were caused by consuming vegetables, such as carrots and iceberg lettuce [52,72]. However, other foods (milk, pork, and water) are suspected sources of *Y. pseudotuberculosis* infections [55]. A *Y. pseudotuberculosis* clonal outbreak in France occurred in 2020, which was caused by the consumption of tomatoes as the suspected source of infection [7]. Therefore, the occurrence of *Y. pseudotuberculosis* may be higher than statistics indicate, especially on organic vegetable and livestock farms where food and farm animals have contact with the outside environment.

**Table 1:** The possible sources and reservoirs of *Yersinia*.

Human Activity	Animal and Plant Sources of *Yersinia* spp.
Food consumption	*Yersinia enterocolitica* Food prepared from raw pork products (the main food source), treated sausage, spinach [59,63]; Chicken [57]; Raw or medium-done pork [58]; Imported fruits and berries [58]; Iceberg lettuce, radicchio rosso [83]; Unpasteurized milk [62,64]; Raw minced pork (young children in Germany) or prepared minced pork in the household [84].
Risky behavior	*Yersinia enterocolitica* Eating in a canteen [58]; Playing in a sandbox [84]; Contact with birds [84].
Possible zoonotic exposure	Association with the backyard slaughter of pigs; Flies carrying antibiotic-resistant *Enterobacteriaceae* strains of the bacteria (Libyan hospitals) [85].
Animal contact	The enteropathogenic serotypes of *Yersinia* include BT2, O:9; BT2 O:5.27; and BT4, O:3 isolated from various animal species. Cattle (fattening pigs)—principal reservoir for human infection [61,86]; The feces of cows, sheep, and goats [62,64]; Red or roe deer, wild boars (isolated the *Ail* gene in rectal swabs—suggestive of virulence gene for *Y. enterocolitica*) [70,86]; Brown hares (European countries) [66]; Wild boars (Spain) [71]; Bats (Germany) [67]; Wild rodents (interspecies carriers between reservoirs in European pig farms) [68]; Flies carrying antibiotic-resistant *Enterobacteriaceae* strains of the bacteria (Libyan hospitals) [85]. Secondary sources Contact with domestic animals [59,62,63].
Human-to-human transmission (possible route)	Rarely transmitted; *Y. enterocolitica* transmitted by a food handler [87]; The nosocomial outbreak of *Y. enterocolitica* diarrheal infection.
Waterborne transmission	The consumption of untreated drinking water (an outbreak of *Y. enterocolitica* due to tap water from a small-scale water system in Japan) [88]; Environmental waters and sewage (pathogenic *Y. enterocolitica* in water sources and sewage in Brazil) [89]; Contact with untreated water, unreticulated sewerage; Environmental water types (reservoirs and creeks).
	*Y. pseudotuberculosis*
Food consumption	This foodborne pathogen is rarely isolated from food. Fresh produce; Vegetable juice [42]; Untreated surface water [42]; Homogenized milk [43].
Outbreaks	Japan [42]; Canada [43]; Europe [6]; Russia [3]; New Zealand [4]; Iceberg lettuce [72]; Carrots [73]; Raw milk [74].

## 7. *Yersinia* Transmission

Both *Y. enterocolitica* and *Y. pseudotuberculosis* are characterized by psychrophilicity—the ability to multiply and accumulate in the environment in low and variable temperatures (4–12 °C) while maintaining or increasing their virulence [3]. Adapting to low temperatures allows bacteria to initiate an infectious process. As a result, their increased motility, chemotaxis, adhesion, invasion, resistance to phagocytosis, and toxin formation are observed. All of them are key factors necessary for the development of an outbreak or an epidemic [3].

*Yersinia* species possess type II and type III secretion systems (T2SS and T3SS), which are involved in initiating host infection. The T2SS is present in all *Yersinia* species, both pathogenic (e.g., the T2SS of the human pathogenic *Y. enterocolitica* strain 1b called Yts1) and nonpathogenic [90]. The T3SS is a hallmark of infection with pathogenic *Yersinia* species and induces Ysc (which forms a multimeric integral membrane complex in the membrane of eukaryotic cells to help Yop translocation) and the Ysa system (which controls Yop secretion in the early stages of infection and invasion of the epithelial M-cells in the Peyer’s patches) [91,92] (Table 2). System T3SSs are contact-activated secretion systems that allow bacteria to inject effector proteins across eukaryotic cell membranes. Upon contact, the secretion apparatus, called the injection complex, exports two types of hydrophobic proteins called translocators (YopB and YopD in *Y. enterocolitica*) and effectors [91].

A crucial role in the pathogenicity of *Y. enterocolitica* is played by chromosomally transferred virulence genes, including *Ail* (attachment invasion locus), *Inv* (invasin), and *Yet* (*Yersinia* stable toxin) [93,94,95]. The adhesin of *Y. enterocolitica* with pleiotropic virulence effects predominantly expressed by *Y. enterocolitica* is *Yersinia* adhesin A (YadA). This protein enables bacterial attachment to host cells and the extracellular matrix (YadA mediates cell adhesion and bacterial translocation to M cells and the colonization of Peyer’s patches) [94]. The major invasion factor of *Y. enterocolitica* is the invasion protein InvA, which induces proinflammatory host cell responses. InvA induces IL-8 synthesis in epithelial cells by engaging beta1 integrins. Inv-induced beta1 integrin signaling involves the small GTPase Rac; the activation of MAP kinases such as p38, MEK1, and JNK; and activation of the transcription factor NF-κB [95]. The attachment and invasion locus (Ail) cooperates with YadA to ensure high serum resistance to *Y. enterocolitica* [93].

*Y. enterocolitica* is divided into six biotypes. Biotype 1A is nonpathogenic, while five other biotypes (1B, 2–5) cause infections in humans and/or animals. Pigs are the main reservoir of bio-serotype 4/O:3 strains. Biotype 4 is most frequently responsible for human infections worldwide. It is usually associated with the serotype O:3 (4/O:3), occurring in pigs that are asymptomatic carriers. The contamination of pork meat often occurs during the evisceration of pigs at slaughter [96]. Most reported cases of yersiniosis in Poland are caused by serotypes 4/O:3, 2/O:9, and 1B/O:8 [69].

Lipopolysaccharide (LPS) is the major component of the outer leaflet of the outer membrane of Gram-negative bacteria, including the genus *Yersinia*. It is responsible for the activation of the host’s innate immune system. The variability of the LPS structure used by Gram-negative bacteria promotes survival, providing resistance to innate immune system components and preventing recognition by TLR4 [97].

LPS’s lipid moiety, called lipid A, is localized in the membrane and serves as an anchor for the rest of the LPS molecule. LPS in the S (smooth) form consists of an external repeating glycan region called the O-specific polysaccharide (OPS), which is often called the O-antigen [8]. The OPS defines the bacteria’s serospecificity and protects bacteria from host defense mechanisms (phagocytosis and complement-mediated killing) [97].

*Y. enterocolitica* O:3 (YeO3) is considered the most common arthritogenic agent that can cause reactive arthritis (ReA) [98]. Bacteria may gain access to blood circulation, grow in the Peyer’s tissue, and be transferred to the joint via plasma or within lymphatic cells [99]. As a result, *Yersinia* infection can cause reactive arthritis, which is associated with the synthesis of specific antibodies.

Unfortunately, some cellular components, such as LPS or endotoxin, persist in the joint (even after bacterial elimination), and they initiate the clinical symptoms [97]. Therefore, the *Yersinia* antigens (including LPS) are detected in synovial fluid cells [99]. In addition to LPS, the complement system is also detected in synovial fluids and plays a significant role in the induction of *Yersinia* inflammation [100]. Interestingly, complement inhibition is associated with a less severe form of the disease [101]. For example, mice deficient in complement components were found to be resistant to arthritis [100].

**Table 2:** Characteristics of the *Yersinia* outer proteins (Yops) and their possible use in clinical practice.

General Characteristics	Crucial Yop Function	Mechanism	Therapeutic Use of the Recombinant Form
YopM			
Molecular weight 41–55 kDa [102]; Connects with PRK and RSK and regulates pro- and anti-inflammatory gene transcription [103]; Colonizes spleen, liver, and lungs [104]; Causes disruption of inflammasome formation [105]; Depletes NK cells [106]; Induces caspase 3-mediated apoptosis [107]; Inhibits apoptosis and migration [108]; ↑ anti-inflammatory IL-10 concentration [109].	A cell-penetrating scaffold protein; An important virulence factor with anti-inflammatory activities.	↓ secretion of pro-inflammatory cytokines: IL-1β, IL-12, IL-15, IL-18, IFγ, and TNF-α.	Involved in the treatment of auto-inflammatory diseases, e.g., Psoriasis; RA; IBD.
		Interacts with mature α-thrombin.	↓ ability of α-thrombin to induce platelet aggregation.
YopE			
A GTPase-activating protein for Rac1 and RhoA; Inhibits the activities of RhoA, Rac1, and Cdc42 (the small GTPase signaling molecules) [110]; Diminishes phagocytosis and Yop translocation; Inhibits IL-8 synthesis, which is an important chemoattractant and activator for neutrophils [111]; ↓ IL-1β maturation [112]; ↓ ROS levels in macrophages—pre-requisite cells during splenic colonization by *Yersinia* [110]; ↓ neutrophil migration, which mainly depends on Rho-mediated signaling [113].	GTPase-activating protein; Plays a major role in the initial bacterial defense against phagocytes [114].	YopE, together with YopT, are inhibitors of caspase-1 activation.	Involved in the treatment of the following caspase-1-related diseases: IBD; Crohn’s disease; RA.
YopT			
A 36 kDa cysteine protease; Induces a phenotype similar to YopE; Cleaves RhoA in vivo [115].	An irreversible inhibitor of Rho-GTPases	↓ inflammation in synovial tissues (through ROK inhibition) [116]	RA—also as a local treatment for inflamed synovial tissues.
		↓ inflammation	Arteriosclerosis; Erectile dysfunction; Neurologic disorders.
YopO			
The anti-phagocytic effector of *Y. pseudotuberculosis* and *Y. pestis*; A protein-kinase-A-secreting virulence factor [117]; Targets Rho-GTPases and Gαq; Prevents phagocytosis of the invading bacteria of host cells; Comprises of three domains (GDI domain, Ser/Thr kinase, membrane localization) [117]; Causes actin depolymerization [118].	Intervenes with the regulation of actin polymerization => disruption of the actin cytoskeleton => impaired bacterial phagocytosis by macrophages [119].	Treatment of diseases associated with hyperactivated Rho-GTPases.	IBD; RA.
		Gαq impairment.	↓ differentiation of Th17 cells and ↑ IBD progression [120].
YopJ/P			
Called YopJ in *Y. pestis* and *Y. pseudotuberculosis* and YopP in *Y. enterocolitica*; Various YopJ/P isoforms responsible for different translocation, substrate binding efficiencies, and virulence [121]; Prevents NF-κB and MAPK activation, inhibition of the inflammatory response, and apoptosis in macrophages and dendritic cells [122].	Has strong anti-inflammatory effects: Induces cell death in macrophages and dendritic cells (only YopP) but not in epithelial or natural killer cells [123]; Inhibits IL-8 and IL-6 [124]; ↓TNF-α signaling and secretion (animal model) [125].	Impacts signaling cascade	Used in psoriasis, RA, IBD, and cancer treatment
		Option for a treatment with bacteria-derived cell-penetrating proteins and topical application	Cell-penetrating proteins used in RA and topical application in psoriasis
		Cell-penetrating YopP has anti-inflammatory effect	↓ TNF-α-induced signaling and secretion triggers apoptosis in activated macrophages synthesizing TNF-α [126]
YopH			
Causes disruption of peripheral focal complexes [127]; YopH has protein tyrosine phosphatase activity with a key function in the blocking of phagocytosis by the pathogen [117]; Impairs T and B cell activation; YopH of *Y. enterocolitica* (but not *Y. pseudotuberculosis*) causes ↓ phagocytosis in murine dendritic cells [127,128]; Inhibits ROS synthesis and the inflammatory response [127]	Enables secretion of a highly potent and versatile phosphotyrosine phosphatase and causes the following: IL-2 secretion and proliferation [129]; IL-8 secretion; Phagocytosis.	Blocks tumor necrosis factor α (TNF-α)	Used in RA (↓ bone erosion, but not inflammation) [126,130]
		Targets p130cas (essential for the actin remodeling of osteoclasts)	↑ osteoclast activity [131]
		↓ activation of the Akt pathway in macrophages (animal models)	Potential use in psoriatic arthritis [132]
		Activates T-cells by monocytes	Causes a persistent inflammatory state in RA [126]
		Injected directly into the tumor	↓ tumor mass or its progression (dose-dependent effect) [133]
		Small molecule inhibitors of YopH	Possible use in the treatment of *Y. pestis* infections [134]
YopQ			
Inhibits detection of the T3SS by the innate immune system and/or by regulating the rate of Yops translocation [135]	The anti-inflammatory effect might also have an impact on host cell proteins like Rac1 [136]	Regulates the translocation rate of Yop effectors into eukaryotic cells [135]	Prevents inflammasome activation.

GDI—guanosine dissociation inhibitors (constitute a family of small GTPases that serve a regulatory role in vesicular membrane traffic); Gαq—heterotrimeric G protein complex; IBD—inflammatory bowel disease; IFγ—interferon-γ; NK—natural killer; PRK or PKN—the serine/threonine protein kinase C-related kinases; RA—rheumatoid arthritis; Rac1—a member of the Rho family of GTPases (intracellular transducers of multiple signaling pathways); Rho family proteins—members of a major branch of the Ras superfamily of small GTPases [a family of small (~21 kDa) signaling G proteins]; ROK—Rho-kinase; ROS—reactive oxygen species; RSK—ribosomal S6 kinase; Ser/Thr—serine/threonine protein kinase; T3SS—type 3 secretion system; TLRs—Toll-like receptors; TNF-α—tumor necrosis factor; Yop—*Yersinia* outer protein.

## 8. Clinical Symptoms

The course of acute infection is usually self-limiting, starting in the intestine and spreading to the mesenteric lymph nodes. However, serious complications are also observed, especially in immunocompromised individuals and elderly patients with multiple comorbidities [84]. Yersiniosis is usually asymptomatic but can cause severe gastrointestinal symptoms (Table 3). Sepsis develops very rarely, especially in people with severe comorbidities, such as immunodeficiencies, diabetes, severe malnutrition, or alcoholism. Sepsis can be complicated by organ abscesses. In patients with genetic predispositions (e.g., HLA-B27 positive), pain in the knee, ankle, and wrist joints may develop a month after the onset of diarrhea. Arthralgia usually spontaneously resolves within six months; however, chronic reactive arthritis may also develop [35]. Incidentally, in people with genetic predispositions, myocarditis or glomerulonephritis may develop. In addition, the asymptomatic carriage of *Y. enterocolitica* is also possible [97,99].

### 8.1. Gastrointestinal Symptoms

*Y. enterocolitica* and *Y. pseudotuberculosis* are enteropathogenic species that commonly cause mild and self-limiting gastrointestinal symptoms. Patients usually report fever, abdominal pain, diarrhea, and occasionally vomiting; rarely, mesenteric lymphadenitis develops [84]. Both infections occur primarily in children but are also reported in adults [13]. Risk factors that increase the likelihood of chronic gastrointestinal symptoms include irritable bowel syndrome, functional constipation, and gastroesophageal reflux disease preceding *Y. enterocolitica* infections [137].

*Y. pseudotuberculosis* can cause severe symptoms, such as fever and acute abdominal pain, which result from inflammation of the mesenteric lymph nodes, and are difficult to distinguish from acute appendicitis [52,72]. *Y. pseudotuberculosis*, being an enteric pathogen, causes gastrointestinal infections mainly in children (usually mild enteritis); however, in the elderly, it can cause severe symptoms leading to septicemia [13]. Both enteropathogenic species can cause serious complications, mainly pseudo-appendicular syndromes, liver or spleen abscesses, and sepsis [9,10,138,139]. Pseudo-appendicular syndromes mimic typical appendicitis [50] and are reported mainly in children [139], whereas sepsis is described in elderly patients [9]. Severe symptoms are observed in adults with multiple comorbidities (e.g., diabetes, cirrhosis) and the geriatric population [13].

### 8.2. Musculoskeletal Symptoms

Extraintestinal complications caused by *Y. enterocolitica* and *Y. pseudotuberculosis* are similar and include symptoms such as reactive arthritis (ReA), erythema nodosum (EN), and conjunctivitis [84]. However, reactive arthritis and erythema nodosum are diagnosed more frequently in *Y. pseudotuberculosis* infections (mainly serotypes O:3) than in *Y. enterocolitica* infections [52,140]. In contrast, infections caused by *Y. pseudotuberculosis* are much less common than intestinal infections and are mainly reported during outbreaks [52,140,141]. ReA is an aseptic and asymmetric arthritis, mainly affecting the joints of the lower limbs, which is usually a self-limiting infection, mainly in susceptible individuals with a positive HLA-B27 antigen [142].

The titer of antibodies against *Y. enterocolitica* detected in synovial fluid correlates with serum concentrations [143]. One useful prognostic marker of ReA is the HLA-B27 antigen. It has been shown that patients with a positive HLA-B27 antigen test result are more likely to develop chronic or severe arthritis than those with a negative antigen test result [144]. Furthermore, the presence of this antigen results in a lower efficacy in eliminating yersiniosis compared to those with a negative HLA-B27 antigen test result [145].

Genetic analyses have shown that *Y. pseudotuberculosis*, which causes FESLF, is the genetic precursor of the plague pathogen Y. pestis. The transition of *Y. pseudotuberculosis* to *Y. pestis* is associated with the acquisition of several genes responsible for the pathogenic properties of this species [46]. FESLF is an acute generalized manifestation of an infectious disease characterized by severe toxic-allergic syndrome caused by repeated bacteremia with relapses and exacerbations of the infection. Predominant damage occurs in the gastrointestinal tract (gastroenteritis, lymphadenitis, appendicitis), liver (hepatitis), and joints [3].

**Table 3:** Clinical characteristics of *Yersinia* spp. infections.

Type of Infection	Mild and Self-Limiting	Severe
Primary (gastrointestinal)	Diarrhea; Abdominal pain in the lower right quadrant (usually in children 5–14 years old); Vomiting [84]; Fever; Conjunctivitis [146].	Pseudo-appendicular syndromes Mimicking appendicitis with a normal appendix in the T1 projection of MRI [138]; Predominantly in older children and young adults [139]. Septicemia Mainly in elderly patients with co-morbidities (hypersideremia, diabetes) [9]. Other Liver or spleen abscesses [10]; Septic arthritis [11]; Cutaneous aseptic abscesses [12].
Secondary	Reactive arthritis Mainly in patients with *Y. enterocolitica* (O:3 and O:9 serotypes); Antibodies against *Y. enterocolitica* detected in synovial fluids [143]; Usually self-limiting polyarthritis; Mostly in susceptible HLA-B27-positive individuals [142]. Erythema nodosum	Rarely chronic reactive arthritis; Mostly in susceptible HLA-B27-positive individuals [142].
*Y. pseudotuberculosis*		
Primary (gastrointestinal)	Mild enteritis Fever; Abdominal pain; Diarrhea; Mainly in children [13]. Acute infection Self-limited; Starts in the intestine (enteritis/gastroenteritis) and spreads to the mesenteric lymph nodes; Acute enteritis/gastroenteritis, mesenteric lymphadenitis [44]; Diarrhea; Fever and abdominal pain [147]; May resemble acute appendicitis [14,44,45].	Acute abdominal pain; Painful pseudo-appendicitis caused by mesenteric lymphadenitis [52,72]. Acute infection or septicemia in the following risk groups The elderly; Patients with multiple co-morbidities such as diabetes and liver diseases (cirrhosis), iron overload [13].
Secondary	Reactive arthritis With polyarticular involvement after an outbreak (caused by *Y. pseudotuberculosis* serotype O:3); Enthesopathy; Mainly in HLA-B27-positive disease (sacroiliitis, painful Achilles tendon, and heel pain) [140]. Non-articular involvement Erythema nodosum; Desquamation; Pneumonia; Nephritis [6].	Recurrent ReA.
FESLF	Severe toxic-allergic syndrome, repeated cyclically (a cyclic course); Gastroenteritis, mesadenitis, appendicitis, hepatitis, pancreatitis; Pneumonia, myocarditis, meningoencephalitis, nephritis; Scarlet-like form and nodular erythema; Arthralgia; Septicemia (lethal outcomes) [3].	Recurrent FESLF [3,46]; Mainly in Russia and Japan [46].

ReA—reactive arthritis; FESLF—severe systemic inflammatory symptoms called Far Eastern scarlet fever.

## 9. Diagnosis

The diagnostic process should be based on clinical evaluation of the current symptoms. It is worth noting that some symptoms are age-specific. For example, gastrointestinal symptoms usually predominate in children, while in adults, in addition to abdominal symptoms, articular symptoms may predominate. However, yersiniosis can occur in different age groups of patients and is detected both in childhood and in old age.

In most cases of infectious diarrhea, *Y. enterocolitica* is not suspected as a potential source of infection. Therefore, the incidence of yersiniosis is largely underestimated, and the difficulty in isolating these pathogens from polycontaminated samples delays the diagnosis [148].

Detection of the organism in a clinical sample is necessary to make a definitive diagnosis of yersiniosis. In clinical practice, isolation of the bacteria from stool, blood, wounds, throat swabs, bile, mesenteric lymph nodes, and cerebrospinal fluid allows for confirmation of the diagnosis [16]. Stool culture is the most readily available method of confirming the diagnosis of *Y. enterocolitica*; however, it should be performed within the first fourteen days of illness. If it is positive, it confirms the infection. Unfortunately, standard stool cultures (without a specific *Y. enterocolitica* incubation growth medium) do not detect the bacilli of *Y. enterocolitica* and are laborious [18].

The most sensitive method is a prolonged low-temperature culture on a selective growth medium [148]. Since *Y. enterocolitica* does not ferment lactose, and is oxidase negative and urease positive, some data suggest direct plating on cefsulodin–irgasan–novobiocin (CIN) agar followed by enrichment procedures according to the reference method ISO 10273:2003 [149]. Unfortunately, even on such a specific medium, *Y. enterocolitica* is rarely detected. For example, in the analysis of 3622 stool samples, a positive test result was observed in seven samples from five patients [150]. Therefore, culturing on CIN or on other specific media that allow for growth at 25 degrees Celsius is not a cost-effective procedure and should therefore not be routinely used.

Another recently described method for detecting Yersinia is the Statens Serum Institut enteric medium (SSI), which allows for the growth of Yersinia at 37 °C. SSI should not be used to detect *Y. pseudotuberculosis* because the growth of this bacterium at 37 °C is inhibited. It is a good method for detecting the pathogenic strains of *Y. enterocolitica* (most nonpathogenic Yersinia species are inhibited on SSI). However, in contaminated human stool, the recovery of *Y. enterocolitica* colonies on SSI at 37 °C is difficult and less sensitive than on CIN at 28 °C. Therefore, despite its limitations, CIN incubated at 28 °C is still required for the successful isolation of enteropathogenic Yersinia from stool [148].

The course of the disease varies from mild to aggressive. If the prodromal symptoms predominate, specific serological tests can help to confirm *Yersinia* infection. These tests are commonly used to detect IgG, IgA, and IgM [151]. Specific antibodies are usually detected in the serum or synovial fluid of patients with the clinical symptoms of yersiniosis using ELISA or immunoblotting. These tests allow for the detection of antibodies against *Yersinia* LPS, ECA-specific antibodies, and *Yersinia*-specific immune complexes [152].

IgM antibodies are markers of the acute phase of yersiniosis, which are synthesized within the first week of infection and reach peak levels in the second week (Table 4). They decline to normal levels within 3–6 months. In rare cases, such as IgM deficiency, antibodies against *Yersinia* in this class are not synthesized [153]. The lack of IgM synthesis is rare (less than 300 cases described in the literature) and is defined as an IgM concentration level lower than 0.40 g/L [154]. Selective IgM deficiency (sIGMD) results from immune deregulation associated with a severe lack of B cell differentiation [155].

IgA antibodies are the most important markers of reactive arthritis. They increase at the onset of yersiniosis and decrease after infection [151]. However, they may persist for 14 to 16 months after the first symptoms, especially in the cases where development of reactive arthritis or chronic enteritis occurs. If the course of yersiniosis is typical (uncomplicated), they can be detected up to five months after infection. IgA is synthesized in the intestine at a rate of 3–5 g/day and initially appears in the blood at a concentration of 2 to 3 mg/mL [121,156]. IgA is essential for the immunological protection of mucosal surfaces in the respiratory and gastrointestinal tract [157]. IgA antibodies to YOPE (23 kDa), YOPD (35 kDa), and YOPB (41 kDa) occur in chronic enteritis, whereas antibodies to YOPD develop in 90% of cases of reactive arthritis. IgA is not synthesized in selective IgA deficiency, the most common type of primary immunodeficiency [158]. However, reduced or absent IgA in the blood may accompany normal levels of IgM and IgG, especially in children over four years of age [158]. IgA antibodies are most important in diagnosing reactive arthritis [151].

In chronic yersiniosis, elevated IgA levels persist with increasing IgG. Interestingly, all patients with positive IgG and IgA against two or more YOPs develop chronic symptoms, often leading to persistent *Yersinia* infection [159]. Analysis of the IgG subclasses (IgG1, IgG2, IgG3, IgG4) in patients with arthritis (n = 35) and uncomplicated yersiniosis (n = 49) showed that in patients with gastritis, the prevalence of IgG2 antibodies to *Yersinia* Yop proteins increased with age and was the highest after the age of 40. The IgG1 corresponding to Yop proteins and IgG3 to LPS were diagnosed more frequently in children than adults [160].

It is worth emphasizing that any antibody analysis should be interpreted cautiously, as cross-reactivity has been observed between *Yersinia* and *Borrelia burgdorferi*, *Brucella*, *Chlamydia pneumoniae*, *Bartonella*, and *Rickettsia*. In addition to reactivity with specific pathogens, they may cross-react with thyroid-stimulating immunoglobulin (TSH) [161].

Serological tests are usually performed in most laboratories, but the presence of antibodies is not part of the diagnostic criteria for yersiniosis. Antibody testing can be used as a screening tool to detect the current infection, but further confirmation is required by another test, most often by stool culture.

Unfortunately, the commonly used procedures are expensive and time-consuming, and therefore are rarely used in standard microbiological laboratories [148]. Therefore, scientists and technologists are looking for procedures that will allow for more accessible isolation of these bacterial enteropathogens. Recently, several multiplex molecular tests have been developed that combine various methods (microscopy, antigen testing, culture, and real-time PCR) and detect Yersinia and other gastrointestinal pathogens (up to 20) directly from clinical stool samples within one hour.

One of the useful tools for rapid and sensitive diagnosis, distinguishing three pathogenic Yersinia groups, is the multiplex PCR method. It enables the differentiation of three pathogenic *Yersinia* groups: highly pathogenic *Y. enterocolitica* (including serotype O8), low pathogenic *Y. enterocolitica*, and *Y. pseudotuberculosis*, due to the unique band patterns for each species from the fecal samples. To establish specific species, four primer pairs are chosen to detect the genes that are responsible for the virulence in pathogenic *Yersinia* species. These genes include *Ail* (attachment invasion locus, present on the chromosome of pathogenic *Y. enterocolitica* strains), gen *FyuA* (ferric yersiniabactin uptake receptor A, present on the chromosomal DNA of highly pathogenic *Y. enterocolitica*), *Inv* (invasion, present on the chromosome of pathogenic *Y. pseudotuberculosis*), and *VirF* (virulence regulon transcriptional activator, encoded on a plasmid of pathogenic *Y. enterocolitica* and *Y. pseudotuberculosis*), which are responsible for the virulence in pathogenic Yersinia species [15]

Since traditional stool cultures are insufficient, many laboratories have recently adopted PCR-based gastrointestinal pathogen panels (GIPP) to increase the speed and efficiency of pathogen detection; for example, commercial multiplex assays such as Verigene EP, FilmArray GI, xTAG GPP, Go-GutDx^®^, and BD MAX^TM^ GIPP have been adopted [16,17,18]. For example, Go-GutDx^®^ is the DNA stool testing kit used for the confirmation of infectious diarrhea, including not only Yersinia, but also other pathogens such as *Clostridium difficile*, *Campylobacter jejuni*, *Salmonella enterica Typhimurium*, *Shigella* spp., *STEC (stx1*, *stx2)*, *Vibrio* spp., and *Yersinia enterocolitica* [17]. In contrast, the BD MAX^TM^ GIPP system detects significantly more *Yersinia enterocolitica* than traditional cultures, with most of these positive results not recoverable by culture. Interestingly, irregular PCR amplification curves occurred if the viable organisms were not recovered in culture, but still yielded positive automated interpretations [18]. The results of nucleic acid amplification interpretation tests for many pathogens must be related to the clinical picture because these tests detect DNA and do not necessarily involve living organisms.

Clinical isolates from Yersinia-infected patients have recently been characterized at the molecular subtyping level using whole genome sequencing (WGS). WGS is a powerful tool for subtyping *Yersinia enterocolitica*. It showed that *Y. enterocolitica* BT 1A strains and BT 4/O:3 strains are genetically diverse, while BT 2/O:3 strains are all ST12 strains and clustered closely [162]. WSG also allows the comparison of the “New World” 8081 strain (1B/O:8), which predominates in the United States (1B/O:8), to the epidemiological pattern in Europe, Japan, and China [biotype 3 strain, 105.5R (r) (O:9) are representatives of the Old World biogroup]. The comparison of the sequenced genome of the mentioned biotypes shows that both strains have more than 14% conservation of specific genes. Many of the loci representing ancestral clusters contributing to enteric survival and pathogenesis are present in strain 105.5R (r) but are lost in strain 8081. The comparative genome analysis indicated that these two strains [biotype 3 strain, 105.5R (r) (O:9) and strain 8081—91B/O:8)] may have attained their pathogenicity by completely separate evolutionary events [163].

The WGS can also be used for food analysis. For example, the study by Stevens et al. shows that the pathogenic *Y. enterocolitica* strains were isolated from 32 of 100 pork samples, from 25 of 100 chicken meat samples, and 22 of 97 produce samples (fresh herbs and salads), all collected at the retail level in Switzerland in 2024. The whole-genome sequencing (WGS) results revealed that three strains belonged to biotype 4 (from pork) and each of them carried a pYV-like plasmid harboring 44 virulence factors. The other 86 strains belonged to biotype 1A, and all isolates belonged to 45 sequence types. Interestingly, plasmids from the same type were identified in different sequence types, showing that genetic exchange between them does occur. Fortunately, none of the isolates harbored plasmid-mediated antimicrobial resistance genes. The *Y. enterocolitica* biotype 4 (n = 3) and biotype 1A (n = 3) were clonal to the *Y. enterocolitica* previously isolated from patients. Thus, *Y. enterocolitica* in food is related to human strains, and the genomic sequences have been adapted to food matrices [162].

The presence of characteristic ultrasonographic findings for Y. enteritis may be helpful in diagnosing *Y. enteritis* infection. Ultrasonographic findings such as enlarged ileocecal lymph nodes, greater wall thickness of the terminal ileum, and presence of a pericecal hyperechoic region are present during infection. The combined presence of a mean ileocecal lymph node major–minor axis ratio < 1.51 and a pericecal hyperechoic region offers 100% sensitivity for *Y. enteritis* compared to other bacterial enteritis [164].

Endoscopy is not warranted in the diagnosis of yersiniosis but is used in the cases of diagnostic uncertainty in which a biopsy may resolve clinical doubts. The *Y. enterocolitica* infection clinically and histopathologically resembles Crohn’s disease (mimicry phenomenon).

Both entities have similar clinical features, often involving the ileocecal region, and histopathological examination may reveal granulomas. However, in the cases of YE infection, central necrosis of the granulomas and a perigranulomatous lymphoid cuff are seen in intestinal biopsy material [165]. Endoscopic findings include round or oval protrusions and shallow and irregular ulcerations scattered in the edematous mucosa in the terminal ileum, without abnormalities in the colon. Ileal ulceration usually appears 4–5 weeks after the onset of symptoms, with ileal perforation occurring five weeks later [166]. Large intestine involvement is rarely reported [167].

**Table 4:** Diagnostic tests used in *Yersinia* infection.

Diagnostic Test	Characteristics	Clinical Meaning
ELISA	Commonly used to detect IgG, IgA, and IgM [151]; Allows for the detection of antibodies against *Yersinia* LPS, ECA-specific antibodies [160], and *Yersinia*-specific immune complexes [152].	Confirms the infection; Useful in final diagnosis, particularly in ReA [151].
Western blot	More sensitive and specific than ELISA; Utilizes YOPs for the detection of *Yersinia* antibodies against *Y. enterocolitica*, *Y. enterocolitica*, and *Y. pseudotuberculosis* [151].	Allows for the differentiation of specific antibody isotypes: Enables differentiation between IgG and IgA antibodies (acute vs. chronic infection) [151]; Enables IgG and IgA detection in patients with IgM deficiency [153].
Endoscopy	Detects changes in terminal inflammation of the ileum, ileocecal valve, and cecum (sometimes ascending colon) [168].	Enables taking a biopsy with an assessment of the degree and type of inflammation [168].
The histological findings of *Yersinia* infection	Not pathognomonic; Patchy villous atrophy and crypt hyperplasia; Mixed acute and chronic inflammation; Focal neutrophilic cryptitis [169]; Epithelial cell granulomas composed of histiocytes, small T-lymphocytes, and plasma monocytes with suppuration of the centers [151].	Histological findings are relatively nonspecific [168]; Usually reflects mixed acute and chronic inflammation with focal neutrophilic cryptitis [169].

ReA—reactive arthritis; SpA—spondyloarthropathy; RA—rheumatoid arthritis; ELISA—enzyme-linked immunosorbent assay; LPS—lipopolysaccharide, ECA—enterobacterial common antigen; GI—gastrointestinal; ReA—reactive arthritis.

## 10. Treatment of *Yersinia* Infection

### 10.1. Treatment of Intestinal Yersiniosis

Mild intestinal ischemia usually resolves spontaneously. Diarrhea requires adequate hydration, electrolytes, and nutrient supplementation (Table 5). Severe and prolonged disease may require antibiotics, primarily quinolones; however, aminoglycosides or tetracyclines in combination with trimethoprim–sulfamethoxazole (TMP-SMX) have also been used [151,170,171,172]. Most organisms are sensitive to aminoglycosides and fluoroquinolones [173].

However, antibiotic treatment is recommended only in severe or complicated diarrheal infections, especially in the case of concomitant or suspected bacteremia or in immunocompromised individuals [171,172]. In specific populations, such as the elderly; diabetics; or those with iron overload, alcoholism, or chronic diseases, treatment should be considered [173]. An example is the use of antibiotics in patients with predominant extramesenteric symptoms such as lymphadenopathy with high fever, weight loss, septic syndrome, and hepatitis. Tetracycline is also used in the treatment of human yersiniosis [170]. Although antimicrobial therapy alleviates symptoms, it does not affect the course of the disease. This fact is confirmed by studies that could not link the duration of symptoms of the enteritis caused by *Y. enterocolitica* infections with antimicrobial treatment [174].

Unfortunately, there are little data on the efficacy of *Y. enterocolitica* treatment; however, available data show that ciprofloxacin has a beneficial influence and causes the disappearance of bacteria from the gut-associated lymphoid tissue compared with placebo. Conversely, patients receiving a placebo have a higher amount of circulating IgA antibodies against YOPs, which are detected for a long time, than patients treated with ciprofloxacin [175]. Similar serologic changes were observed in the case of *Y. enterocolitica*-induced reactive arthritis, which is characterized by persistent serum IgA antibodies against YOPs compared to patients with uncomplicated infections. Therefore, it is suspected that prolonged antibiotic administration could eliminate persistent bacilli from the gut, thus eliminating the circulating IgA antibodies from the serum [151]. Moreover, successful ciprofloxacin monotherapy resulted in a serologic switch, causing the prolonged presence of IgG antibodies against YopD in serum (rarely, antibodies against YopH and YOPM are observed). Due to the elimination of the infection trigger by ciprofloxacin, antibody synthesis is stopped, preventing the development of chronic infection. Unfortunately, the high levels of antibiotic resistance in *Y. enterocolitica* may result in ineffective prevention and treatment of this disease in humans and animals, which may lead to serious threats to public health [176].

**Table 5:** Treatment of yersiniosis.

	Management	
Stage of the Disease	Management	Characteristics
acute severe Yersiniosis	Supportive care	Fluids; Electrolytes; Nutritional restoration.
	Hospital admission and antibiotics	Elderly people; Immunocompromised patients; Diabetic patients; Patients with iron metabolism disorders: iron overload, deferoxamine therapy (used in iron overload therapy); Alcohol addicts; Patients on chronic hemodialysis; Patients undergoing deferoxamine therapy; Chronically ill patients with many co-morbidities.
	Abdominal abscess	Surgical draining
	Appendicitis suspicions	Surgical exploration (appendicitis and pseudoappendicitis are difficult to distinguish based on clinical symptoms or imaging studies) If the appendix is normal, lymph nodes should be removed for histological examination
	Antibiotics	Quinolones [175]; Aminoglycosides; Tetracyclines +/− TMP-SMX; Third-generation cephalosporins +/− TMP-SMX [177].
	The acute phase of ReA	Ciprofloxacin 500 mg twice daily orally compared with placebo for three months causes faster remission and pain relief compared with the group without antibiotics [177].
Chronic infection	Intestinal infection	Mild to severe infection; Abdominal pain located on the right side of the abdomen and fever, diarrhea, and vomiting, rarely acute inflammation of the intestinal lymph nodes in adults [84]; Low-grade fever, crampy abdominal pain, loose stool with mucus or blood, and vomiting in acute enteritis in young children (with the risk of dehydration); Symptoms usually appear four to seven days after infection and last from one week to three weeks or longer [84].
	ReA	Rarely synthesized antibodies to YopH and no occurrences of antibodies to YopM (YopH and YopM are produced at the late stage of the disease and reflect the more chronic phase); The disappearance of IgA associated with the disappearance of *Yersinia* from intestinal biopsies in *Yersinia*-associated SpA [177]; The prolonged administration of antibiotics could eliminate persistent bacilli from the gut, thus eliminating the circulating IgA antibodies from the serum [177].
Preventive measures	Education and healthy behavior	Hand washing; Meticulous food handling; Education, especially for patients who have experienced previous infections of *Y. enterocolitica* [178]; The elimination of contact between food handlers, healthcare workers, or childcare workers and healthy people until they are symptom-free for 48 h [5]; Information about the possibility of bacteria in fresh vegetables (e.g., lettuce) after seven days of refrigerated storage (4 °C), which cannot be disinfected by washing with tap water or water with 60 ppm chlorine added [178].

TMP-SMX—trimethoprim–sulfamethoxazole; SpA—spondyloarthropathy.

### 10.2. Treatment of Skin and Mucous Membrane Lesions

Mild skin lesions usually do not require treatment. In more severe lesions, keratinolytic agents (e.g., topical salicylic preparations), glucocorticosteroids(GCSs), or calcipotriol in the form of a cream or ointment can be used. In severe skin lesions, methotrexate or retinoids are usually necessary. In the case of balanitis cricoid, topical treatment with a weak GCS (e.g., topical hydrocortisone cream) is used. The treatment of uveitis requires the use of mydriatics and steroids in the form of eye drops. In severe cases, if the lesions do not resolve, an oral GCS may be necessary.

### 10.3. Treatment of Reactive Arthritis

Since ReA is an aseptic arthritis, it does not require antibiotic treatment and is usually self-limiting. Recent data show that antibiotic therapy is indicated only if there is a documented active infection, mainly in chlamydial infection. However, in active yersiniosis, the implementation of ciprofloxacin results in the shortening of symptoms [175]. Joint pain may well respond to NSAIDs or glucocorticosteroids, but chronic ReA may require the introduction of disease-modifying antirheumatic drugs (DMARDs). The drug used as the first choice is sulfasalazine [179,180].

## 11. Prevention

For many years, the factors that have the greatest impact on public health have been analyzed in order to develop control strategies. Unfortunately, the quality of available information on the risks of *Yersinia* spp. is low. Implementation of good practice guidelines throughout the vegetable production chain could prevent the presence of identified risks. Solid epidemiological studies on foodborne illnesses potentially associated with vegetable consumption are necessary [181].

An effective approach to this zoonosis prevention and control that integrates animal, human, and environmental ecological principles is required [182]. Risk analysis uses predictive models, which are powerful tools for public health and epidemiology, predicting the spread and spillover of pathogenic bacteria based on external environmental factors like temperature, precipitation, altitude, and vegetation distribution, as well as the characteristics of isolates like serotypes and biotypes, and the monitoring and alerting of relevant zoonotic diseases. In the case of *Yersinia*, the unifactor and multifactor analyses performed by Liang et al. showed that the distribution of *Y. enterocolitica* in animals is influenced by environmental factors such as altitude, mean temperature, and precipitation [183]. The temperature (12.70 °C) and precipitation (1.34 mm) of the collection locations were closely related to the pathogenic isolates [184].

The preventive measures should include handwashing after exposure to an infected animal and safe food processing (both domestic and industrial) [185]. It is advisable to avoid the consumption of raw pork and its products [186]. Routine water treatment and disinfection are obligatory. Since Yersinia can be preserved in blood, the screening for this pathogen in blood and blood products is obligatory [187]. Considering the numerous factors related to food processing as well as environmental factors, it is necessary to develop new food safety strategies to prevent and control *Y. enterocolitica* infections [188].

## 12. Conclusions

*Yersinia* infections are widespread in most countries with a cold climate and are considered an important causative agent of sporadic and epidemic human enteric disease, less frequently ReA. The complex mechanisms involved in the initiation and transmission of the infection in host cells, such as T3SS, LPS, endotoxin, and Yop proteins, initiate host infection, leading to human gastroenteritis. Bacteria are transmitted via the fecal–oral route, most often after consuming contaminated animal products (e.g., raw pork, unpasteurized milk). Diagnosis is often difficult and requires confirmation of the infection by various bacteriological (the isolation of *Yersinia* from biological samples) and serological methods (e.g., the detection of antibodies in blood serum or synovial fluid). Ultrasound or CT might be necessary in the case of pseudoappendicitis, and colonoscopy in the suspicion of ulcerative colitis. Yersiniosis is usually mild and self-limiting, but in elderly and immunocompromised patients, it can be a cause of severe sepsis or pseudo-appendicular syndromes. *Y. pseudotuberculosis* infection is rare, and mainly sporadic cases are reported, but it can also cause epidemic outbreaks.

Although recent data on *Yersinia* infections are published every year, there are areas that require further analysis and development. First, the available data on *Yersinia* food poisoning are usually underestimated. Therefore, the meticulous reporting of infections will enable the development of accurate epidemiological data. Secondly, uniform standards of pathogen identification are still lacking, and data on the effectiveness of treatments are very limited. Therefore, more effective screening of infections and analysis of currently available antimicrobial therapies for their efficacy is advisable. Thirdly, it is also important to understand the precise mechanisms and complications associated with chronic infection, which allows for better adjustment of treatment. Fourthly, it is crucial to educate patients and food handlers, report infections, and implement risk assessments through hazard analysis. Fifthly, the development of vaccines will facilitate the elimination of the disease in the most prevalent regions.

## Abbreviations

*Ail*	attachment invasion locus
ECA	enterobacterial common antigen
FESLF	Far Eastern scarlet fever
GDI	guanosine dissociation inhibitors (which constitute a family of small GTPases that serve a regulatory role in vesicular membrane traffic)
Gαq	heterotrimeric G protein complex
IBD	inflammatory bowel disease
HPI	high-pathogenicity island
IFγ	interferon-γ
inv	invasin
LPS	lipopolysaccharide
NK	natural killer
OPS	O-specific polysaccharide
PRK (PKN)	the serine/threonine protein kinase C-related kinases
RA	rheumatoid arthritis
Rac1	A specific member of the Rho family of GTPases (intracellular transducer of multiple signaling pathways)
Rho family proteins	A member of a major branch of the Ras superfamily of small GTPases [a family of small (~21 kDa) signaling G proteins]
RCTs	randomized control trials
ReA	reactive arthritis
ROK	Rho-kinase
ROS	reactive oxygen species
RSK	ribosomal S6 kinase
Ser/Thr	serine/threonine protein kinase
sIGMD	selective IgM deficiency
T3SS	type 3 secretion system
TLRs	Toll-like receptors
TMP-SMX	trimethoprim–sulfamethoxazole
TNF-α	tumor necrosis factor
TSH	thyroid-stimulating immunoglobulin
yet	*Yersinia* stable toxin
Yop	*Yersinia* outer protein
YPM	*Y. pseudotuberculosis*-derived mitogen
